# Assessment of Bisphenol A (BPA) Exposure in Dairy Cows Using Hair Samples Analysis

**DOI:** 10.3390/ani15070939

**Published:** 2025-03-25

**Authors:** Slawomir Gonkowski, Manolis Tzatzarakis, Nariste Kadyralieva, Elena Vakonaki, Thomas Lamprakis, Ismail Sen, Askarbek Tulobaev, Fatih R. Istanbullugil, Aidai Zhunushova, Liliana Rytel

**Affiliations:** 1 Department of Clinical Physiology, Faculty of Veterinary Medicine, University of Warmia and Mazury in Olsztyn, Oczapowskiego 13, 10-957 Olsztyn, Poland; slawomir.gonkowski@uwm.edu.pl; 2 Laboratory of Toxicology, School of Medicine, University of Crete, 70013 Heraklion, Greece; tzatzarakis@uoc.gr (M.T.); evakonaki@gmail.com (E.V.); lamprakist@gmail.com (T.L.); 3 Department of Histology and Embryology, Faculty of Veterinary Medicine, Kyrgyz-Turkish Manas University, Bishkek 720042, Kyrgyzstan; nariste.kadyralieva@manas.edu.kg; 4 Department of Internal Medicine Faculty of Veterinary Medicine, Kyrgyz-Turkish Manas University, Bishkek 720042, Kyrgyzstan; ismail_sen@manas.edu.kg; 5 Department of Basic Science, Faculty of Veterinary Medicine, Kyrgyz-Turkish Manas University, Bishkek 720042, Kyrgyzstan; askarbek.tulobayev@manas.edu.kg; 6 Department of Food Hygiene and Technology, Faculty of Veterinary Medicine, Kyrgyz-Turkish Manas University, Bishkek 720042, Kyrgyzstan; fatih.ramazan@manas.edu.kg; 7 Department of Pharmacology and Toxicology, Faculty of Veterinary Medicine, Kyrgyz-Turkish Manas University, Bishkek 720042, Kyrgyzstan; ayday.cunusova@manas.edu.kg; 8 Department of Internal Diseases with Clinics, Faculty of Veterinary Medicine, University of Warmia and Mazury in Olsztyn, 10-720 Olsztyn, Poland

**Keywords:** dairy cows, bisphenol A, endocrine disruptors, exposure

## Abstract

Bisphenol A (BPA) is a plasticizer known to have various adverse effects on humans and animals. Exposure of farm animals to BPA negatively impacts their health and poses a threat to humans who consume products of animal origin. Despite the importance of studying the exposure of farm animals to BPA, the knowledge in this area is quite limited. To date, the assessment of BPA levels in dairy cows using analysis of hair samples has not been performed. The study utilized liquid chromatography-mass spectrometry to analyze hair samples collected from 48 dairy cows in the following regions of Kyrgyzstan: Sokuluk, Alamedin and Ysyk Ata. BPA levels above the limit of detection (LOD = 4.8 pg/mg) were noted in 18.8% of analyzed samples. BPA levels in individual samples ranged from below LOD to 89.1 pg/mg with a median value of 9.3 ± 19.7 pg/mg. The study indicates that dairy cows are exposed to BPA, and hair samples can be used for BPA biomonitoring in farm animals. The present study is the first investigation of BPA levels in cows’ hair, which is one of the best matrices to study long-term exposure of living organisms to environmental pollutants. Additionally, this study is the first to analyze BPA in biological samples from Kyrgyzstan.

## Simple Summary

Bisphenol A (BPA) is an organic substance commonly used in the plastic industry. It is present in many everyday items. BPA may penetrate the environment and living organisms. It is known that BPA exhibits toxic properties and may negatively affect various internal organs. The current understanding of farm animal exposure to BPA is very limited. In the present study, hair samples collected from dairy cows were used for the first time to test for BPA content. The results revealed the presence of BPA in the collected samples, confirming that farm animals are indeed exposed to this compound. It should be noted that farm animal exposure to BPA may not only harm animal health but may also pose risks to humans who consume products of animal origin.

## 1. Introduction

Farm animals are exposed to environmental pollutants that can negatively impact their health and potentially contaminate animal products, posing risks to human health [1,2]. One hazardous substance that commonly pollutes the environment and shows multidirectional toxic activities is bisphenol A (BPA) [3]. BPA is commonly utilized as a plasticizer in various industries and can be found in many everyday items, such as bottles, food containers, furniture, clothes, epoxy resins, electronic components and household items [3,4]. It is estimated that the current global production of BPA is approximately 10 million metric tons [4].

The popularity of BPA originates from its presence in plastics that are known for durability, plasticity and resistance to high temperatures [3]. BPA can migrate from the plastics and penetrate food, drinking water and the environment, and this process is influenced by various factors, including temperature, pH, lipid content, the presence of some substances (e.g., alcohol), mechanical damage of plastics containing BPA, and the duration of contact between the plastics and food or the environment [3]. The presence of BPA has been detected in surface water, soil, air and dust worldwide [3,4]. BPA may also enter human and animal bodies, and its presence has been identified, among others, in blood, urine, breast milk, semen and amniotic fluid [3,5,6,7].

BPA (bisphenol A) is known to have multiple adverse effects on living organisms. These harmful effects are associated with BPA’s influence on estrogen, androgen, aryl hydrocarbon and peroxisome proliferator-activated receptors [3]. As a result, BPA is classified as an endocrine disruptor. Its effects on organisms primarily manifest as disturbances in the hormonal system, where it impairs processes regulated by sex hormones, insulin, leptin and adiponectin. Consequently, BPA affects various systems in the body, including the reproductive, nervous, cardiovascular, immune and digestive systems [3,5]. Previous studies have also shown proinflammatory, genotoxic, mutagenic and carcinogenic properties of BPA. Additionally, there is a connection between BPA exposure levels and the risk of infertility, hypertension, and neurodegenerative diseases [3,5,8]. Due to its significant harmful effect on the human body, BPA is restricted under law regulations in various countries. European Union legislation introduced on 19 December 2024 under the Classification, Labeling and Packaging (CLP) Regulation banned the use of BPA in food contact materials. This means that this substance will no longer be allowed in the production of drink bottles, cans and other food containers [9].

Currently, many methods are used to determine BPA content in biological materials, food and elements of the natural environment. One of them is an immunological method, most often performed using commercial ELISA tests. While this method is quick, inexpensive and easy to make, it has a few drawbacks. Despite efforts to introduce modifications for improvement, it still exhibits relatively low sensitivity [10,11], and therefore, it is not recommended for the determination of BPA in biological samples [10].

Chromatographic techniques, including liquid chromatography (LC), high-performance liquid chromatography (HPLC), ultra-high performance liquid chromatography (UHPLC), single-step solid phase extraction procedure followed by high-performance liquid chromatography (SPE-HPLC), isotope-dilution liquid chromatography (ID-LC) or gas chromatography (GC), are more widely used in determining BPA levels in biological samples [10,12,13,14,15,16]. It should be noted that chromatography may be connected with various detectors, including mass spectrometer (MS), tandem mass spectrometer (MS/MS), electrospray tandem mass spectrometer (ESI/MS/MS), ultraviolet-visible detector (UV-VIS), fluorescence detector (FLD), diode array detector (DAD), charged aerosol detector (CAD) and evaporative light-scattering detector (ELSD) [16,17,18,19,20,21]. From the above-mentioned techniques, LC or UHPLC with mass spectrometry or tandem mass spectrometry is the most commonly used in the analysis of BPA in biological samples, including hair [16,18,22].

It should be underlined that in addition to studies on BPA levels in living organisms, some studies describe levels of BPA metabolites, especially BPA-glucuronide (BPA-G). BPA-G is the main BPA metabolite formed under the influence of UDP-glucuronosyl-transferase in the intestine and liver and excreted with urine [23]. Previous studies have shown that the analysis of BPA-G levels in human urine and, to a slightly lesser extent, in blood serum may be suitable for BPA biomonitoring in the human population [24,25,26,27]. However, limitations in the use of analysis of BPA-G levels in biomonitoring studies are related to the rapid elimination of BPA from the organism and significant interspecies differences in BPA metabolism. Moreover, there is a lack understanding regarding the exact metabolism of BPA in various species of wild and domestic animals [23,24].

Contrary to humans, knowledge of the exposure of domestic animals to BPA is relatively limited. Some studies have proven that dogs and cats are significantly exposed to this substance, primarily due to their close proximity to humans [28,29]. In the case of livestock, the majority of previous studies on BPA levels focus on the presence of this substance in processed, commercial products of animal origin, which are considered potential sources of human exposure to BPA [30,31,32]. However, only a few studies have evaluated the intravital exposure of farm animals to BPA and the levels of this substance in their bodies [33,34]. Research on cattle has identified the presence of BPA in the serum of buffaloes [35] and bovine urine, feces and raw milk [36,37,38].

According to the best knowledge of the authors, there have been no investigations assessing the long-term exposure of cows and other farm animals to bisphenol A (BPA) using hair samples. It should be noted that among various matrices used for biomonitoring BPA exposure, hair samples are gaining prominence [16,39]. This is because of the advantages of this matrix. Specifically, hair samples are easy to collect and store. Previous studies have demonstrated that the analysis of hair samples can yield results comparable in sensitivity, repeatability, and reliability to those obtained from blood serum or urine, although the levels of substances in the hair are not strictly correlated with their levels in body fluids and tissues [39,40]. Moreover, hair samples appear to be the most effective matrix for studying long-term exposure to environmental pollutants, as substances accumulate in the hair and do not fluctuate rapidly in concentration, unlike in urine or blood serum [39].

Given the above, the aim of the present study was to investigate, for the first time, the long-term exposure of dairy cows to BPA using a non-invasive method of BPA analysis in hair samples. Additionally, the study sought to determine whether hair analysis is a useful tool for assessing the exposure of dairy cows to BPA. Furthermore, this is the first biomonitoring study on BPA and its presence in biological samples from Kyrgyzstan.

## 2. Materials and Methods

### 2.1. Reagents

In the present study, the following reagents were used: BPA (≥98%) from Sigma-Aldrich (St. Louis, MO, USA); methanol (LC-MS grade) from Fischer Chemicals (Loughborough, UK); phenobarbital (internal standard—IS) from Lipomed AG (Arlesheim, Switzerland); ultrapure water, obtained using a Merck Direct-Q 3UV water purification system (Darmstadt, Germany).

### 2.2. Sample Collection

The study included 48 adult dairy cows of various breeds (Kyrgyz, Alatau, Holstein and Swiss), aged from 3 to 8 years. Hair samples were collected in April and May 2023 from animals on farms located in three regions of Kyrgyzstan around Bishkek—the capital and largest city of the Kyrgyzstan Republic. The selected regions, i.e., Sokuluk, Alamedin, and Ysyk Ata, were chosen due to their proximity to Bishkek, which is characterized by a relatively high level of environmental pollution, high industrialization and urbanization. The vicinity of the city may influence the occurrence of anthropogenic pollutants in the rural areas of the mentioned regions, leading to the exposure of farm animals to such pollutants. Additionally, these regions serve as a key agricultural base for Bishkek, supplying animal products to the city’s residents. They are also one of the most agriculturally developed regions of Kyrgyzstan. Characterization of regions included in the study is presented in Supplementary Materials Table S1.

Samples of approximately 2 g of hair were collected from each animal. For each animal, the hair was cut as close to the skin as possible from the dorsal area (above the shoulder blades). Immediately after collection, contaminations were removed from the hair mechanically, and the hair was washed in methanol and dried. The hair samples were then wrapped in aluminum foil and stored in a dark place at room temperature until further analysis was performed. During the collection procedure, hair samples were not in contact with plastics and other items that may contain BPA. Since the collection of hair samples was painless and non-stressful and performed during routine care or breeding activities, the approval of an ethics committee was not required under the current laws of Kyrgyzstan or the European Union. Consent to take the samples was obtained from animal owners.

During the study, special attention was paid to avoiding extrinsic contamination of samples with BPA. Throughout the sampling and storage procedure, hair samples were not in contact with plastic items, which may contain BPA. Only glass vials were used for the extraction of the hair samples. Vials had previously been washed with ultrapure water and LCMS grade methanol and dried at 150 °C for 2 h in order to remove any possible contamination. Moreover, the extraction solvent was analyzed to investigate any presence of BPA and blank hair samples were analyzed in each batch to examine any carry-over effect.

### 2.3. BPA Extraction

The hair was cut into small fragments using metal scissors measuring several millimeters in length. To remove external contaminations, the hair was rinsed four times: twice with ultrapure water and twice with methanol and dried at 50 °C according to the method described by Tzatzarakis et al. [18]. The efficacy of the rinsing was verified by analyzing selected final wash solutions for the presence of BPA, which provided negative results.

The extraction was performed in accordance with the method described by Tzatzarakis et al. [18]. In summary, 100 mg of the hair with 2 × 2 mL of methanol and 25 ng IS were placed in glass screw tubes and extracted in an ultrasonic water bath with continuous ultrasonic waves for 2 × 2 h, with periodic mixing with a vortex system. The temperature was monitored to ensure it did not exceed 50 °C. The obtained extracts were then combined and evaporated to dryness under nitrogen steam at 35 °C. After adding 100 μL of methanol to the residues, the solution was transferred into 2 mL vials with inserts for liquid chromatography-mass spectrometry (LC-MS) analysis and 10 μL of the solution was injected into the system.

### 2.4. Instrumentation

The analysis was performed with an LC-MS system (Shimadzu, Kyoto, Japan, 2010 EV) and a Supelco Discovery column C18 (250 mm, 4.6 mm, 5 μm; Sigma-Aldrich, St. Louis, MO, USA) at 30 °C. The analysis was conducted with a flow rate of 0.6 mL/min using a water gradient as solvent A and methanol as solvent B, starting at 15% of solvent B (0–1 min), 98% B (linear ramp) (1–18 min), stable at 98% (18–22 min) and finally 15% B (22–27 min). To monitor BPA, an atmospheric pressure chemical ionization (APCI) and a quadrupole mass filter in negative selected ion monitoring (SIM) mode were used with ions m/z 227.15, 259.1 for the BPA and 231.05 for the IS. The interface, CDL, and heat block temperatures were set at 400 °C, 200 °C, and 200 °C, respectively; the detector voltage at 1.5 kV; and the nebulizing gas flow at 2.5 L/min. Data acquisition and processing were performed using LC–MS Solution software (Shimadzu, Version 3). The total time of analysis was 27 min.

### 2.5. Method Validation

The efficacy of the methods used was evaluated through various analytical parameters. Standard solutions (0, 25, 50, 100, 250, and 500 ng/mL) of the analyte were made in methanol, and the linearity was found to be 0.9975 (*n* = 4). Spiked sample (0, 25, 50, 100, 250, and 500 pg/mg) analysis was performed for the construction of the spiked curves with linearity of 0.9963 (*n* = 4) and used for the calculation of BPA levels in authentic samples (Supplementary Materials Figure S1).

Both the limit of detection (LOD) and limit of quantification (LOQ) were evaluated using the signal-to-noise ratio (s/n > 3 and s/n > 10) and calculated at 4.8 and 16.0 pg/mg using the lowest spiked level (25 pg/mg), (*n* = 3), respectively. These findings align with values reported in previous studies (2.9 and 9.7 pg/mg, 2.6 and 8.5 pg/mg, respectively) [18,41].

To evaluate recovery, accuracy, and inter-day precision (%RSD) of the method, spiked blank cow hair samples that had previously been analyzed and provided negative results for BPA were used. The mean % recovery and accuracy values of the method were determined at 93.1% (*n* = 4) and 92.7% (*n* = 4), respectively, while the corresponding mean %RSD was 19.8% (*n* = 3) for the spiked levels of 25, 50, 100, and 500 pg/mg of hair (Table 1). Furthermore, blank tests (extracted and concentrated pure solvent) were performed to ensure there were no contaminations or carry-over effects during the extraction and analysis procedure.

### 2.6. Statistical Analysis

The statistical analysis was conducted using GraphPad Prism version 9.2.0 (GraphPad Software, San Diego, CA, USA). The obtained data were analyzed using descriptive statistics with the calculation of the following values: arithmetic mean ± standard deviation (SD), geometric mean ± geometric SD factor, and median. A non-parametric Kruskal–Wallis test was used to determine the differences between BPA levels in cows from different regions. Differences were considered statistically significant at *p* < 0.05. BPA concentrations lower than LOD were included in the statistics as LOD/2 (2.4 pg/mg).

## 3. Results

The present study found BPA concentration levels above LOD (4.8 pg/mg) in 18.8% of the analyzed hair samples (Table 2). Among the samples with BPA levels exceeding the LOD, their values fluctuated from 16.1 pg/mg to 89.1 pg/mg. The mean (±SD) BPA concentration amounted to 9.27 ± 17.72 pg/mg, and the median value was lower than LOD (Table 2).

The highest frequency of BPA presence above the LOD was found in the Sokuluk region, where six samples (31.1% of all samples) were recorded with BPA levels ranging from 19.2 pg/mg to 89.1 pg/mg. In the Alamedin region, two samples had BPA levels surpassing the LOD (13.3% of all samples) with concentrations of 16.1 pg/mg and 38.6 pg/mg. In the Ysyk Ata region, BPA levels above LOD were noted only in one sample (5.9% of all samples from this region) (Table 2).

The mean BPA concentration (±SD) (considering values below LOD as LOD/2) amounted to 18.78 ± 26.98 pg/mg, 5.73 ± 9.76 pg/mg and 3.44 ± 4.27 pg/mg in the Sokuluk, Alamedin and Ysyk Ata regions, respectively. Confidence intervals at a confidence level of 95% were 18.78 ± 13.22 [5.560–32.000] in the Sokuluk region, 5.73 ± 4.94 [0.791–10.669] in the Alamedin region and 3.44 ± 4.27 [1.410–5.470] in the Ysyk Ata region.

**Figure 1:**
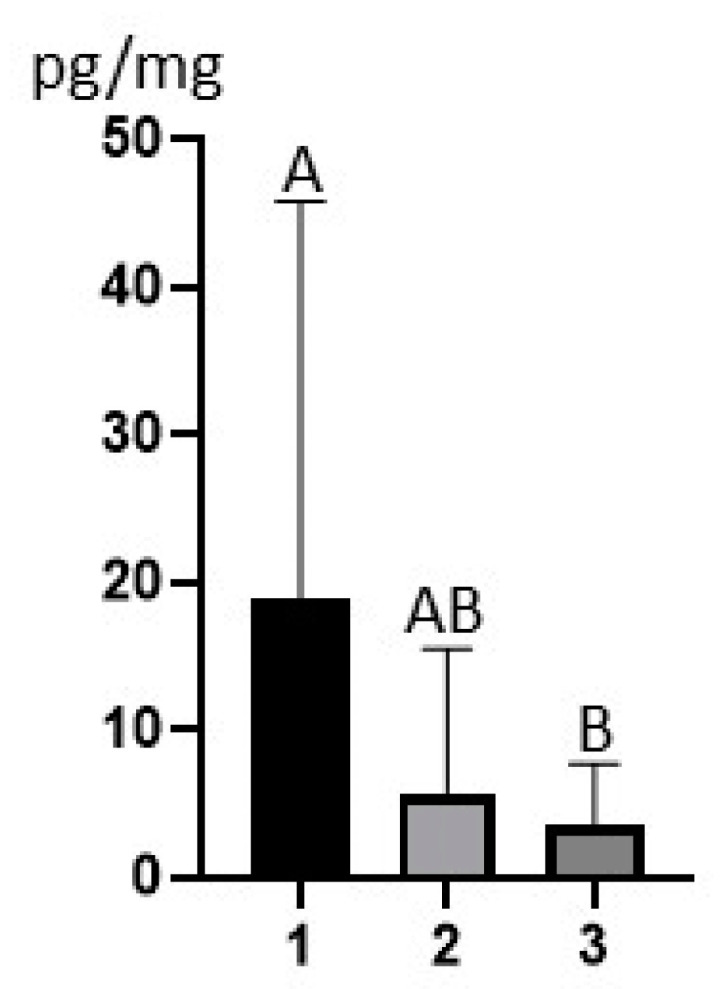
Mean concentration levels (±SD) of bisphenol A (BPA) in the hair samples of dairy cows bred in Sokuluk (1), Alamedin (2) and Ysyk Ata (3) districts. Values that are not statistically significantly different are indicated with the same letter, values that differ statistically significantly (*p* < 0.05) are indicated with various letter. Statistically significant differences were visible between 1 and 3 (*p* = 0.0473) Differences between 1 and 2 (*p* = 0.1963) and between 2 and 3 (*p* > 0.9999) were not statistically significant). The figure was created using GraphPad Prism version 9.2.0 (GraphPad Software, San Diego, CA, USA).

Statistically significant differences were found in BPA mean concentrations between samples collected from cows in the Sokuluk and Ysyk Ata regions (*p* = 0.0473). There were no statistically significant differences between the Sokuluk and Alamedin regions (*p* = 0.1963) and between the Alamedin and Ysyk Ata regions (*p* > 0.9999) (Figure 1).

## 4. Discussion

The present results, which demonstrate the presence of BPA in hair samples collected from dairy cows, strongly suggest that the hair is suitable for research on the exposure of farm animals to this substance. Previous studies have shown that BPA and other environmental pollutants tend to accumulate in hair, which makes their levels less susceptible to short-term fluctuations, unlike in urine or blood, in which levels of BPA may undergo short-term changes. Previous studies on humans have reported that levels of BPA and its analogues in hair are not always closely correlated with BPA levels in serum or urine [39,42,43]. This is completely understandable if we take into account the nature of the matrix, and differences do not mean that one of the matrices is worse than the other. We can imagine such a situation: a person or an animal ate food with low BPA contamination for a long period (e.g., a month or longer). Two days before sample collection for BPA level determination, the individual started eating food or drinking water with clearly higher levels of this compound. A urine or blood analysis will show high levels of BPA. However, hair examination will show a low concentration of this compound. Such a situation results from the fact that hair samples have a larger monitory window (counted in months) in comparison to urine and blood (counted in days or even hours) [39]. Of course, hair as a matrix has some disadvantages. One of them is the fact that substances penetrate hair both through blood vessels and hair bulbs and directly from the external environment, and during analysis, separation of these two penetration routes is impossible [39].

Nevertheless, despite this disadvantage, hair samples are one of the best matrices for studying long-term exposure in both humans and animals [16,29,39]. Simultaneously, the hair sample collection is easy, non-invasive and stress-free (which is important when conducting research on animals). Despite these undoubted advantages, studies analyzing BPA levels in hair samples remain relatively scarce. The majority of them concern humans, and only a few studies have been performed on dogs and wild animals (Table 3).

The comparison of the current results on BPA levels in the hair with the results obtained in previous studies (Table 3) presents certain challenges. First, the previous investigations were performed in different regions of the world, and it is commonly known that environmental pollution of BPA and, hence, the exposure of living organisms to this substance depends on many local factors, including not only industrialization and urbanization of the area but also, for example, the lifestyle and habits of its residents [3,5]. Secondly, the majority of previous studies on BPA in the hair were conducted on humans, and it is logical that humans are more exposed to anthropogenic environmental pollutants (including BPA) than other organisms. This higher exposure is largely due to human-specific practices, such as the use of everyday objects made of plastic at home and in the workplace, such as clothes, and consuming food packaged in plastic containers and bottled water [3,5]. A similar situation is observed in the case of companion animals that live in close proximity to humans and are affected by the same environmental factors [28,29].

Therefore, it is no surprise that the BPA levels noted in the present study were generally lower than those noted in humans in various parts of the world. Moreover, the frequency of detection higher than LOD in humans in the majority of previous studies amounted to 100% (Table 3), while the present study achieved only less than 20%. The frequency of detection as well as BPA levels noted in the dairy cows in Kyrgyzstan were also lower than values observed in dogs, in which BPA at levels above LOD were found in above 90% of samples, and the maximum concentration levels amounted to 436 pg/mg [29]. Interestingly, both the frequency of BPA presence and its concentrations recorded in the current study were significantly lower than those noted in wild animals in Europe. For instance, BPA was found in more than 80% of wild boars and in 100% of Baltic seals, with maximum concentration levels of 508.7 pg/mg and 137.3 pg/mg, respectively [41,50]. This suggests that environmental contamination with BPA is significantly higher in Europe than in Kyrgyzstan. The differences between the current study and previous research on wild animals may originate from various factors influencing BPA exposure. For wild animals, exposure primarily occurs due to environmental pollution, including BPA in surface waters and food sources, as well as their dietary habits. In contrast, farm animals could be exposed to BPA through industrial feed (which may become contaminated during production or through packaging), grazing fields (due to agricultural practices), drinking water, or dust in barns. Additionally, farm animals may come into contact with BPA during breeding and veterinary treatments. The results suggest that farm animals’ exposure to BPA is lower than that of wild animals. As mentioned above, lower farm animal exposure to BPA in comparison to wild animals (seals and wild boars) may result from generally higher environmental pollution in the regions where wild animals were studied, i.e., Europe. This may be connected with a higher degree of urbanization and industrialization and a higher percentage of agricultural areas. Previous studies have reported relatively high levels of BPA in the European environment [54,55]. However, differences may also result from other factors. Seals, in which BPA levels were higher than those noted in the present study, are water organisms which mainly feed on fish, and it is known that levels of BPA in both seawater and wild fish are high (in the water even to 1600 ng/L and in fish even to 13,000 ng/g) [56]. Thus, BPA content in wild fish is much higher than in commercial cattle feed (174.7 ng/g) or feed additives (44.2 ng/g) [35].

In turn, wild boars are animals that increasingly often live near human settlements, visit even big cities, and feed near landfills or cultivated fields [57]. Moreover, wild animals drink surface water that is more contaminated with BPA than tap or groundwater, which is most often used for watering farm animals [58]. The reason for the observed differences in BPA levels between cows and wild animals may also be connected with the intraspecies differences in BPA metabolism, which is not fully explained in cows, seals or wild boars.

Nevertheless, the presence of BPA in the hair samples indicates the long-term exposure of dairy cows to this substance, which constitutes a potential risk to both animal health and the safety of consumers of the products of animal origin.

The number of previous studies regarding the exposure of cows to BPA is relatively low, and they were performed in other parts of the world (mainly in China) and on matrices different from hair (Table 4).

BPA concentration levels in other matrices are not closely correlated with those noted in the hair [39], and the exact metabolism of BPA in ruminants is unknown. Moreover, it is known that exposure of living organisms to BPA varies significantly in different parts of the world and depends on many local factors (including, among others, industrialization and urbanization). Due to these two facts (other matrices and regions), comparing the present results with those of previous studies on BPA in cows is impossible. Nevertheless, previous results (Table 4) and the present study indicate that cows may be exposed to BPA; the degree of exposure depends on the studied area, and there are notable differences in BPA levels among individual animals. The presence of BPA in farm animals indicates that this substance pollutes the environment not only in highly urbanized and industrialized regions but also in rural areas, which confirms previous research [64]. One of the main causes of the occurrence of BPA and other anthropogenic pollutants in rural environments is the irrigation of agricultural lands with water reclaimed from wastewater [42].

The sources of cattle exposure to BPA are not yet fully understood. Previous studies on this issue are extremely scanty. However, it is known that feed and feed additives might be a significant source of cow exposure to BPA. The presence of BPA has been detected in Italy in over 50% of cattle feed samples in concentration levels from 1.2 ng/g to 174.7 ng/g (mean 12.5 ng/g) and in almost 90% of feed additive samples in concentration levels from 4.1 ng/g to 44.2 ng/g (mean 18.3 ng/g) [35]. Another study performed in China has reported that mean BPA concentration levels in cow food amounted to 41.1 ± 11.4 ng/L and suggested that cow food is contaminated with BPA mainly during production, packaging and storage [60]. On the other hand, other studies have shown relatively low mean levels of BPA in grains and by-products (0.52 ng/g) and forages (0.96 ng/g) [65].

Knowledge about other potential sources of BPA in cows is not quite clear. Such a source may be drinking water because it is known that both surface water and tap and groundwater (to a lesser extent) may be contaminated with BPA [58,66]. In contrast, the only previous publication on BPA in drinking water for cattle did not detect the presence of BPA [35]. Cow exposure to BPA probably may also be connected to indoor dust and breeding equipment. Previous studies have indicated that indoor dust mainly contains relatively high levels of BPA [67], but indoor dust in cowsheds has not been studied. Similar studies on the direct impact of the use of breeding equipment on BPA levels in cows do not exist. However, observations showing clearly higher levels of BPA in processed cow’s milk in comparison to raw milk strongly suggest that equipment used for breeding and/or milk collection may be the source of BPA [62].

In the present study, differences in BPA levels in the hair samples between particular regions were visible. Moreover, the SD of mean BPA levels from samples collected in the Sokuluk region was significantly higher than those noted in other regions. This is connected with the fact that in this region, BPA in levels above LOD was found in the largest percentage of samples, and BPA levels in particular animals were varied. In the Alamedin and Ysyk Ata regions, a greater number of samples contained BPA at concentrations below the LOD. For statistical purposes, BPA concentrations lower than LOD were treated as a value LOD/2, which resulted in a decrease in SD.

The lowest BPA concentrations were found in animals from the Ysyk Ata region. This finding may be attributed to the fact that this region is located farthest from the center of Bishkek among the studied regions and is characterized by the lowest population density (Supplementary Materials Table S1). This is all the more likely because BPA is an anthropogenic environmental pollutant, and the positive correlation between a high degree of industrialization and urbanization and BPA levels in the environment is relatively well-known [3,5]. Moreover, previous studies have shown that BPA levels in the hair of people living in rural and less urbanized areas are lower than in humans from cities [18]. This situation also applies to wild boars. The levels of BPA in the hair of animals living near more urbanized and industrialized areas were higher than those noted in animals from areas with low human population density and no industrial activity [41]. Therefore, it can be assumed that such relationships also apply to BPA levels in cow hair. However, without comprehensive research including cows from areas with varying degrees of urbanization and industrialization, this cannot be clearly confirmed.

Another significant issue is the impact of the cow’s exposure to BPA on the content of this substance in animal-derived products, which poses a potential risk for consumers. Previous studies have described the presence of BPA in cow milk, but the majority of them concern processed packed milk and milk products, in which BPA levels may be relatively high and amount to 521 µg/L [62,68,69]. Knowledge about BPA in raw milk is more limited (Table 4). However, BPA levels noted in the raw milk are clearly lower than those noted in processed milk and do not exceed 3 µg/L [1,37,62]. This fact indicates that the contamination of cow milk with BPA is connected with the production process of milk for consumption. However, although BPA levels in the raw milk were relatively low, the consumer exposure, calculated considering a hypothetical raw milk consumption level, may be above the newest tolerable daily intake (TDI) recommended by the European Food Safety Authority (EFSA) (0.2 ng/kg bw/day) [1,9,37,62]. However, exceeding the TDI dose requires relatively high raw milk consumption. As it has been calculated, the consumption of 500 mL of raw milk per day may result in a human exposure of 18 ng/day [1]. A similar situation occurs in the case of meat. Most previous studies on BPA levels concern processed meat and meat products [31,70]. However, there are also studies suggesting in-life contamination of farm animal muscles with BPA [31,34]. A mean level of BPA in beef steak (not collected immediately after the animal death, but bought in the shop) amounted to 2.93 µg/kg (i.e., equivalent to 2.93 ng/g) [70]. This shows that daily consumption of such meat may result in exceeding the TDI for BPA established by EFSA. Therefore, BPA in raw milk and raw meat may have an influence on human health. This is all the more likely because long-term exposure to even small doses of BPA may result in a negative impact on processes occurring in the human body [71,72]. Unfortunately, due to a lack of adequate studies, it is impossible to determine how the BPA levels observed in hair during the present study translate into BPA content in animal products.

Similar difficulties apply to determining the adverse impact of the BPA amounts observed in this study on cow’s health. However, this impact is supported by the fact that even small doses of BPA disturb the functions of internal organs in cows and other farm animal species [73,74,75]. The majority of previous studies on BPA’s impact on cows concern changes in reproductive organs and have been performed in vitro. Among others, it is known that BPA causes abnormal expression of key miRNAs during oocyte maturation, changes in viability and steroid production of theca cells, induces apoptosis and oxidative stress reactions in granulosa cells, as well as influences embryo development [74,76,77,78,79,80]. Other in vitro studies on bovine tissues have described significant genotoxic effects of BPA on lymphocytes and BPA-induced changes in adrenal medullary cells [81,82]. It should be pointed out that the above disturbances may appear even under the influence of low doses of BPA [74], which suggests that BPA levels noted in the present study may also negatively impact animal health. However, to date, there are no studies on the impact of cow exposure to BPA on milk production or epidemiological works examining the relationship between animal exposure to BPA and health status. This is due to the technical difficulties of the above-mentioned research, such as, among others, the choice of an appropriate, easily accessible matrix reflecting long-term exposure to BPA. Therefore, the present results may contribute to the development of such research because they showed that hair samples, which are easy to collect and store, are suitable for the evaluation of BPA levels in cows.

The present study has certain limitations. The first limitation is the relatively small number of samples included in the study, which may suggest that results are not representative of all cow populations in regions. This limitation results from the reluctance of animal owners to consent to the collection of samples, even though the collection was completely stress-free and painless. However, it should be pointed out that some previous studies on BPA in the hair samples of humans and other species have been performed on an even smaller number of samples (from 5 to about 50—see Table 3). Moreover, previous studies on BPA levels in cows (in urine, feces or milk) usually have also been performed on an extremely small number of samples (Table 4). Only three previous studies on BPA levels in cows have been performed on a larger number of samples than the current study. Therefore, the present study does not differ in terms of number of samples from most studies on the same issue (Table 3 and Table 4).

Another limitation concerns the lack of determination of potential sources of exposure of dairy cows to BPA, determination of BPA levels in feed, water, environment and breeding equipment used in cows included in the study, as well as lack of assessment of the impact of BPA on the cows’ health and milk production. Moreover, the influence of time of year on BPA levels in cow hair was not taken into account in the present study. On the other hand, all samples included in this study were collected at one time of year, and differences in BPA levels between regions were not seasonal. All of the above-mentioned issues are very important and require further comprehensive studies.

Nevertheless, the results of the present study, which for the first time show BPA in biological samples collected in Kyrgyzstan, as well as present long-term exposure of dairy cows to this substance, are interesting and enrich knowledge about BPA in farm animals. Moreover, the present study can be treated as a first step toward further comprehensive studies on the exposure of farm animals to BPA and the impact of this substance on animal health and productivity.

## 5. Conclusions

In conclusion, the present study is the first description of BPA in the hair of dairy cows and the first study on BPA in Kyrgyzstan. The investigation shows that hair is a suitable matrix for studying the long-term exposure of cows to BPA. The presence of BPA in levels above LOD has been found in a relatively small percentage of the analyzed samples (below 20%). The occurrence of BPA in cows’ hair is lower compared to data from humans and dogs in other regions of the world. This could be attributed to two reasons: the generally low environmental pollution with BPA in Kyrgyzstan or the low exposure of dairy cows to this substance. The exposure of cows to BPA may result in the deterioration of animal health and the presence of BPA in products of animal origin, which poses a potential risk to consumers. However, the present study has scientific limitations due to the lack of identification of the source of dairy cow exposure to BPA and the correlation between BPA levels and animal health status, including hematological and biochemical blood parameters. Furthermore, this study did not investigate the effect of BPA on milk production and quality. Therefore, further comprehensive studies are necessary to determine the issues mentioned above, which are of great importance in dairy cow breeding.

## Figures and Tables

**Table 1:** Validation parameters of the applied methods.

	BPA	No of Repetitions
Mean % recovery ± SD	93.1 ± 17.9	*n* = 4
Mean % accuracy ± SD	92.7 ± 7.1	*n* = 4
Precision (%RSD)	19.8	*n* = 3
LOD (pg/mg)	4.8	*n* = 3
LOQ (pg/mg)	16.0	*n* = 3
r^2^ (standard curves)	0.9963	*n* = 4
r^2^ (spiked curves)	0.9975	*n* = 4

BPA—bisphenol A; LOD—limit of detection; LOQ—limit of quantification.

**Table 2:** Bisphenol A concentration (pg/mg) and frequency of BPA presence (%) in hair samples collected from dairy cows included into the study.

Individual Data								
Sokuluk Region			Alamedin Region			Ysyk Ata Region		
Sample No	Age of Animal	BPA Levels	Sample No	Age of Animal	BPA Levels	Sample No	Age of Animal	BPA Levels
1	5	25.0	17	4	38.6	32	4	<LOD
2	6	<LOD	18	7	<LOD	33	4	<LOD
3	4	19.2	19	6	<LOD	34	4	<LOD
4	8	25.6	20	4	<LOD	35	4	20.0
5	4	<LOD	21	6	<LOD	36	4	<LOD
6	6	<LOD	22	4	16.1	37	4	<LOD
7	4	57.4	23	5	<LOD	38	4	<LOD
8	6	89.1	24	6	<LOD	39	4	<LOD
9	3	<LOD	25	5	<LOD	40	4	<LOD
10	3	<LOD	26	7	<LOD	41	4	<LOD
11	3	<LOD	27	6	<LOD	42	4	<LOD
12	3	<LOD	28	4	<LOD	43	4	<LOD
13	3	60.2	29	5	<LOD	44	4	<LOD
14	3	<LOD	30	4	<LOD	45	4	<LOD
15	3	<LOD	31	6	<LOD	46	4	<LOD
16	3	<LOD				47	4	<LOD
						48	4	<LOD
Cumulative data (from all regions studied)								
Range	Arithmetic mean ± SD		Geometric mean ± geometric SD factor		median		Frequency (above LOD)	
<LOD-89.1	9.3 ± 19.7		3.9 ± 2.9		<LOD		18.8	

LOD—limit of detection (4.8 pg/mg).

**Table 3:** Overview of previous studies on bisphenol A in hair samples.

Species	Country	*n*	Method	BPA Concentration Levels (pg/mg)	Reference
Human	Belgium	114	UPLC-MS/MS	<LOQ-587.1	[22]
	China	204	UPLC-MS/MS	5.47–596	[44]
	France	311	UPLC-MS/MS	17.1–1398	[44]
	France	5	UPLC-MS/MS	0.273–7.636	[45]
	Greece	69	LC-MS	13.1–192.8	[18]
	Greece	122	LC-MS	2.6–205.5	[46]
	Greece	100	LC-MS	9.6–650.3	[47]
	Korea	10	LC-ESI/MS/MS	17–22.9	[19]
	Luxembourg	16	LC-MS/MS	<LOD-95.8	[48]
	Luxembourg	264	LC-MS/MS	62.34–35,856.1	[49]
	Poland	42	HPLC	26.1–1498.6	[50]
	Poland	25	LC-MS	3.6–52.9	[51]
	Spain	6	LC-MS/MS	9.2–45	[52]
	Spain	6	LC-MS/MS	24–158	[53]
	Spain	42	LC-MS/MS	24.4–1427	[16]
Dog	Poland	30	LC-MS/MS	<LOD-36	[29]
Baltic seal	Poland	17	HPLC	<LOQ-137.2	[50]
Wild boar	Poland	54	LC-MS	<LOD-508.7	[41]

LOQ—limit of quantitation; LOD—limit of detection; *n*—number of individuals included into the study.

**Table 4:** Overview of previous studies on bisphenol A concentration in pg/mg (indicated by *) or ng/mL (indicated by #) in the cattle.

Country	Matrix	*n*	BPA Levels	Reference
China	feces (milking cows)	10	ND-106.3 *	[59]
China	feces (milking cows)	9	50.5–72.0 *	[60]
China	feces (milking cows)	6	2.3–2.7 *	[36]
China	feces (beef cattle)	6	3.3–4.1 *	[36]
China	urine (milking cows)	6	0.353–0.413 #	[36]
China	urine (beef cattle)	6	1.950–2.120 #	[36]
China	urine (beef cattle)	8	2.3–8.4 (5.4) #	[38]
China	urine (milking cows)	6	2.9–6.9 (5.4) #	[38]
China	raw milk	50	<3.1–13.74 *	[61]
Italy	raw milk	8	<0.1–2.833 #	[62]
Italy	raw milk	72	0.035–2.776 #	[37]
Italy	raw milk	22	ND-2.34 #	[1]
Italy	raw milk (bufalloes)	46	0.5–5.6	[35]
Italy	Blood serum (bufalloes)	190	0.16–6.39	[35]
Poland	raw milk	5	<0.19–<0.64 #	[63]

## Data Availability

All data are available in the manuscript and Supplementary Materials.

## Supplementary Materials

The following supporting information can be downloaded at: https://www.mdpi.com/article/10.3390/ani15070939/s1, Figure S1: Chromatograms of spiked solution of BPA at 0 pg/mg (A) and at 100 pg/mg (B) (RT = 16.7 min for internal standard and 20.13 min for BPA); Table S1: Regions included into the study.
