# Supplementary material for: Assessment of Bisphenol A (BPA) Exposure in Dairy Cows Using Hair Samples Analysis
Source: Animals (Basel). 2025 Mar 25;15(7):939. doi: 10.3390/ani15070939 (PMC11988172; doi:10.3390/ani15070939)
Supplement: Supplementary file 1 [file animals-15-00939-s001.zip › animals-3477307-supplementary.pdf]

Supplementary Materials

Assessment of Bisphenol A (BPA) Exposure in Dairy Cows Using Hair Samples Analysis

Slawomir Gonkowski [1], Manolis Tzatzarakis [2], Nariste Kadyralieva [3], Elena Vakonaki [2], Thomas Lamprakis [2], Ismail Sen [4] , Askarbek Tulobaev [5], Fatih R. Istanbullugil [6], Aidai Zhunushova [7] and Liliana Rytel [8,*]

[1] Department of Clinical Physiology, Faculty of Veterinary Medicine, University of Warmia and Mazury in Olsztyn, Oczapowskiego 13, 10-957 Olsztyn, Poland.
[2] Laboratory of Toxicology, School of Medicine, University of Crete, 70013 Heraklion, Crete, Greece.
[3] Department of Histology and Embryology, Faculty of Veterinary Medicine, Kyrgyz-Turkish Manas University, Bishkek 720042, Kyrgyz Republic
[4] Department of Internal Medicine Faculty of Veterinary Medicine, Kyrgyz-Turkish Manas University, Bishkek, Kyrgyz Republic
[5] Department of Basic Science, Faculty of Veterinary Medicine, Kyrgyz-Turkish Manas University, Bishkek, Kyrgyz Republic
[6] Department of Food Hygiene and Technology, Faculty of Veterinary Medicine, Kyrgyz-Turkish Manas University, Bishkek, Kyrgyz Republic
[7] Department of Pharmacology and Toxicology, Faculty of Veterinary Medicine, Kyrgyz-Turkish Manas University, Bishkek, Kyrgyz Republic
[8] Department of Internal Diseases with Clinics, Faculty of Veterinary Medicine, University of Warmia and Mazury in Olsztyn, Olsztyn, Poland.
* Correspondence: e-mail: liliana.rytel@uwm.edu.pl

Table S1. Regions included into the study.

Region name	Sokuluk	Alamedin	Ysyk Ata
Human population	147,208	158,137	131,503
Human population density (persons/km^2)	92	62	54.5
Administrative center	Sokuluk	Lebedinovka	Kant
The places of sampling - names of village/distance in the straight line from the center of Bishkek (km) / coordinates	Sarban / 10.3/ 42° 47′ 03.5″ N 74° 30′ 48.2″ E Kashka-Besh/8.6/ 42° 48′ 49.3″ N 74° 30′ 26.3″ E	Kyzyl-Birdik / 18.5/ 42° 42′ 49.7″ N 74° 42′ 47.6″ E Tash-Moinok /15.7/ 42° 44′ 21.9″ N 74° 39′21.4″ E	Kant /23/ 42° 53′ 00.2″ N 74° 50′ 39.5″ E

A)

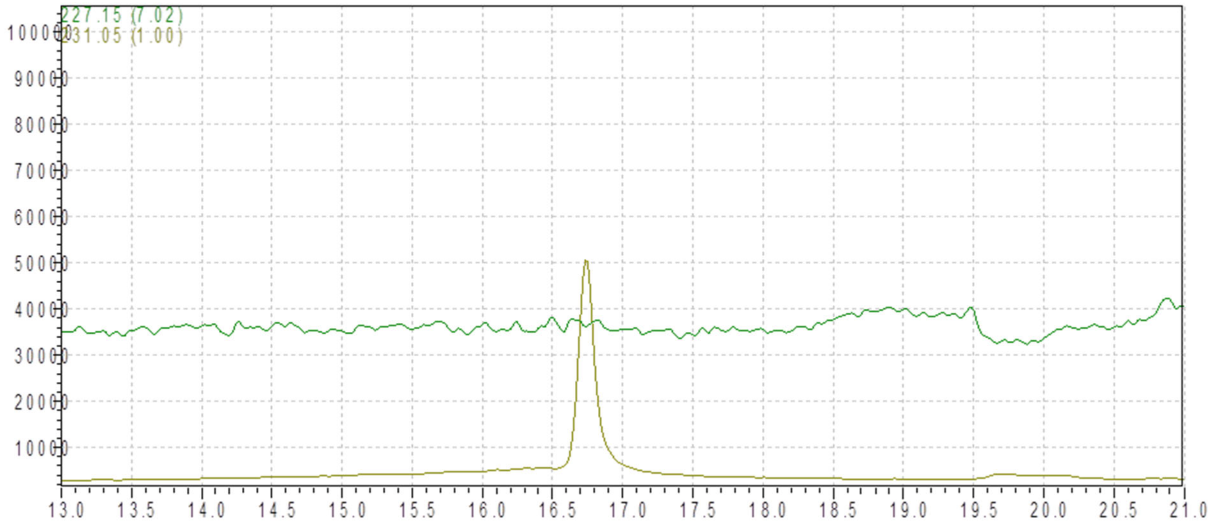

B)

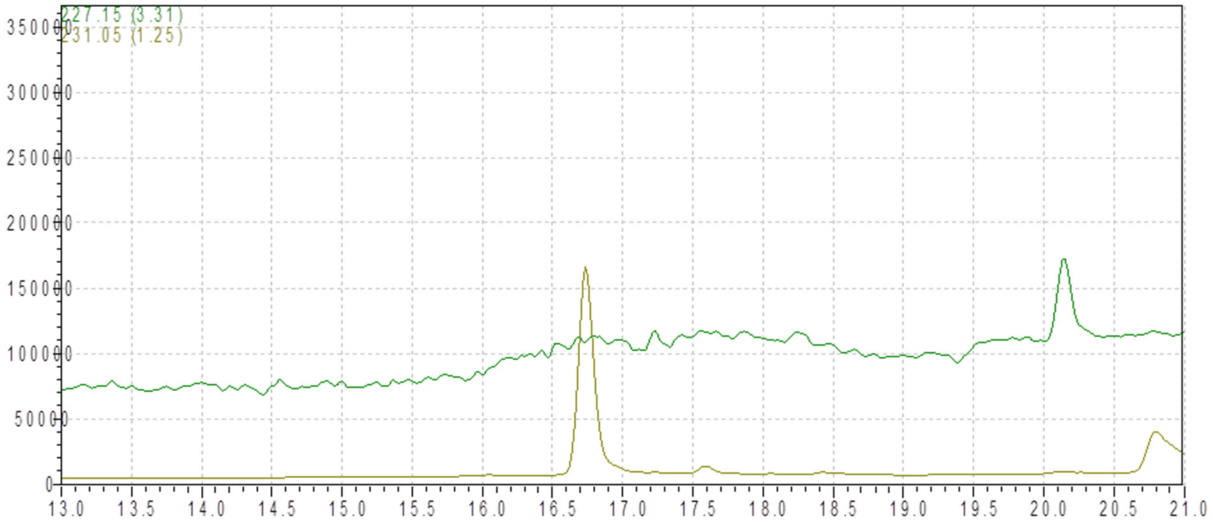

Figure S1. Chromatograms of spiked solution of BPA at 0 pg/mg (**A**) and at 100 pg/mg (**B**) (RT=16.7 min for internal standard and 20.13 min for BPA).
